# Anti-Tumour Necrosis Factor Therapy for Dupuytren's Disease: A Randomised Dose Response Proof of Concept Phase 2a Clinical Trial

**DOI:** 10.1016/j.ebiom.2018.06.022

**Published:** 2018-07-06

**Authors:** Jagdeep Nanchahal, Catherine Ball, Dominique Davidson, Lynn Williams, William Sones, Fiona E. McCann, Marisa Cabrita, Jennifer Swettenham, Neil J. Cahoon, Bethan Copsey, E. Anne Francis, Peter C. Taylor, Joanna Black, Vicki S. Barber, Susan Dutton, Marc Feldmann, Sarah E. Lamb

**Affiliations:** a Kennedy Institute of Rheumatology, Nuffield Department of Orthopaedics, Rheumatology and Musculoskeletal Sciences, Oxford, UK; b Edinburgh Department of Plastic Surgery, St John's Hospital, Livingston, UK; c Oxford Clinical Trials Research Unit, Centre for Statistics in Medicine, Nuffield Department of Orthopaedics, Rheumatology and Musculoskeletal Sciences, Oxford, UK

**Keywords:** Dupuyten's disease, Anti-TNF, Fibrosis, Adalimumab, Myofibroblast

## Abstract

**Background:**

Dupuytren's disease is a common fibrotic condition of the hand that causes irreversible flexion contractures of the fingers, with no approved therapy for early stage disease. Our previous analysis of surgically-excised tissue defined tumour necrosis factor (TNF) as a potential therapeutic target. Here we assessed the efficacy of injecting nodules of Dupuytren's disease with a TNF inhibitor.

**Methods:**

Patients were randomised to receive adalimumab on one occasion in dose cohorts of 15 mg in 0.3 ml, 35 mg in 0.7 ml, or 40 mg in 0.4 ml, or an equivalent volume of placebo in a 3:1 ratio. Two weeks later the injected tissue was surgically excised and analysed. The primary outcome measure was levels of mRNA expression for α-smooth muscle actin (*ACTA2*). Secondary outcomes included levels of α-SMA and collagen proteins. The trial was registered with ClinicalTrial.gov (NCT03180957) and the EudraCT (2015-001780-40).

**Findings:**

We recruited 28 patients, 8 assigned to the 15 mg, 12 to the 35 mg and 8 to the 40 mg adalimumab cohorts. There was no change in mRNA levels for *ACTA2, COL1A1, COL3A1* and *CDH11.* Levels of α-SMA protein expression in patients treated with 40 mg adalimumab (1.09 ± 0.09 ng per μg of total protein) were significantly lower (*p* = 0.006) compared to placebo treated patients (1.51 ± 0.09 ng/μg). The levels of procollagen type I protein expression were also significantly lower (*p* < 0.019) in the sub group treated with 40 mg adalimumab (474 ± 84 pg/μg total protein) compared with placebo (817 ± 78 pg/μg). There were two serious adverse events, both considered unrelated to the study drug.

**Interpretation:**

In this dose-ranging study, injection of 40 mg of adalimumab in 0.4 ml resulted in down regulation of the myofibroblast phenotype as evidenced by reduction in expression of α-SMA and type I procollagen proteins at 2 weeks. These data form the basis of an ongoing phase 2b clinical trial assessing the efficacy of intranodular injection of 40 mg adalimumab in 0.4 ml compared to an equivalent volume of placebo in patients with early stage Dupuytren's disease.

**Funding:**

Health Innovation Challenge Fund (Wellcome Trust and Department of Health) and 180 Therapeutics LP.

## 1 Introduction

Dupuytren's disease (DD) is a common fibrotic disease confined to the hand that affects approximately 4% of the general UK and US populations [1]. The early stages of the disease are manifest as nodules that are typically quiescent for a period and then become active, progressing to cords and flexion deformities of the fingers in approximately 50% of patients [2] and result in loss of hand function [3]. Whilst the mainstay of treatment remains surgical excision (fasciectomy) of the diseased tissue or cords [4], approximately 40% of patients in the USA are treated by disruption of the cords using collagenase or needle fasciotomy [5]. Generally patients undergo these treatments when digits are flexed to 30° or more and hand function is impaired [6]. The recurrence rate following surgery is 21% within 5 years [7] and these individuals may require more extensive surgery involving excision of the diseased tissue and overlying skin (dermofasciectomy). Post-operatively, some patients require prolonged hand therapy and splintage. Complications occur in approximately 20% of patients undergoing surgery [8]. Alternative, less invasive techniques to disrupt the cords with a needle or collagenase digestion are associated with rapid recovery of hand function with minimal therapy. However, recurrence rates are high, affecting 85% of patients treated with percutaneous needle aponeurotomy [7] and 32% of those treated with collagenase [9] at 5 years. The complication rate is 20% following needle aponeurotomy [8] and over 70% after collagenase injection, the majority being minor and mostly transient [10].

The ideal therapy would be directed towards patients with early stage disease to prevent progression to development of cords and subsequent flexion contractures of the digits. Our systematic review [11] highlighted the lack of robust evidence for treatments proposed for early stage DD for which there is currently no approved therapy. Studies reporting the efficacy of intranodular injection of steroids or radiotherapy are limited by a lack of quality, with no blinding or randomisation and the use of subjective outcome measures [11]. Fifty percent of patients receiving steroid therapy developed transient subcutaneous atrophy or depigmentation. Approximately 20–30% of patients receiving radiotherapy developed long-term adverse effects, including dry skin, desquamation, skin atrophy, telangiectasia, erythema, and altered heat and pain sensation. A recent trial reported that injection of collagenase resulted in nodules becoming smaller and softer, with approximately 50% of patients experiencing bruising and pain [12]. Therefore, there is a need to develop an effective therapy to retard progression of early DD and also prevent the development of recurrent disease following surgery, needle fasciotomy or collagenase injection in patients with established finger contractures.

The cell responsible for deposition of the excessive collagenous matrix and contraction in all fibrotic conditions, including DD, is the myofibroblast [13, 14], which is characterised by the expression of α-smooth muscle actin (α-SMA) [15]. Unlike the fibrotic diseases of visceral organs such as the kidney, lung and liver, tissue from patients with DD is readily accessible, allowing identification of potential novel therapeutic targets [16]. Using surgically excised tissue from patients we found that the myofibroblasts in DD are aggregated in nodules in the vicinity of the affected joints, and nodules were absent in patients with more advanced stage disease [17]. Interspersed through the nodule are immune cells, including macrophages, T cells and mast cells, and the nodular cells secrete a variety of cytokines [18]. Comparison of the effects of each of these cytokines showed that only tumour necrosis factor (TNF) converted palmar fibroblasts from patients with DD into myofibroblasts at the low concentrations seen *ex vivo,* but not non-palmar fibroblasts from the same patients or palmar fibroblasts from normal individuals. In contrast, TGF-β indiscriminately converts all fibroblasts into myofibroblasts [18]. This is important as the fibrotic process seen in DD is limited to the palm of genetically susceptible individuals. Genome-wide association studies have highlighted the role of Wnt signalling in DD [19, 20] and we found that TNF acted via the Wnt signalling pathway only in palmar dermal fibroblasts from patients with DD [18]. Myofibroblasts from DD showed a dose-dependent reduction in contractility on treatment with anti-TNF, with a concomitant reduction in expression of α-SMA [18]. All the clinically approved anti-TNF agents assessed were effective in down regulating DD myofibroblast contractility *in vitro*, with the two fully human IgG molecules, adalimumab and golimumab being the most efficacious at the doses tested [18].

Here we report the effects of injection of escalating doses of adalimumab or corresponding volume of placebo directly into the nodules of patients who then underwent surgery two weeks later. Markers of myofibroblast phenotype and collagen production were assessed in the excised samples.

## 2 Methods

### 2.1 Study Design and Patients

Repurposing anti-TNF for Dupuytren's disease (RIDD) is a two-part phase 2 randomised, double-blinded placebo-controlled dose response study to assess the efficacy of local injection of adalimumab in patients with DD. The protocol was reviewed by the South Central Oxford B Research Ethics Committee (Reference number 15/SC/0259) and the Medicine and Healthcare products Regulatory Authority (EudraCT no: 2015-001780-40) and has been published [21] (details of subsequent amendments in appendix). The phase 2a dose escalation study reported here was performed at a single centre in the UK at the Edinburgh Department of Plastic Surgery at St John's Hospital, NHS Lothian. Patients referred to the hand surgery service at St John's Hospital by their general practitioner with a diagnosis of DD were screened for entry to the trial. Eligibility criteria included no prior treatment for Dupuytren's disease to the affected hand, a clinically distinct nodule of DD, suitability for injection of adalimumab and fasciectomy. All potential participants were screened for TB, HIV, hepatitis B and C using serological testing and chest X-ray in accordance with local standard procedures for anti-TNF screening.

During the study a new preparation of adalimumab became available with 40 mg formulated in 0.4 ml. The lower volume and absence of excipients including citrate was expected to result in reduced pain and improve participant acceptability. Therefore, the trial design was modified to include a cohort at a dose of 40 mg using this preparation. The 40 mg in 0.4 ml formulation is currently only available in a pre-filled single use syringe and so only the full syringe dose of 40 mg could be administered with this formulation. Therefore, the trial design was modified to include a cohort of 40 mg in 0.4 ml. Since publication, the protocol has been amended to introduce an endpoint to assess whether adalimumab affects the healing of the surgical incisions or subsequent scarring. This visual assessment of the surgical wounds was carried out by blinded review of the hand photographs taken at baseline and then at 2 and 4 weeks post-surgery.

### 2.2 Procedures

Patients were randomised (3:1) to receive either adalimumab or saline in 3 dose cohorts (15 mg in 0.3 ml, 35 mg in 0.7 ml), both using the 40 mg in 0.8 ml preparation, or 40 mg in 0.4 ml using the more recently introduced formulation, or an equivalent volume of placebo (normal saline) on one occasion by intra-nodular injection two weeks (±3 days) prior to scheduled surgery. Nodule hardness was measured using a durometer (RX-1800-00, Rex Gauge Company, Illinois, USA) and nodule size was assessed using an ultrasound scan before injection and again before surgery. Blood was collected at baseline and again immediately before surgery, and the serum stored at -80 °C prior to analysis. ELISA kits from Promonitor were used according to the manufacturer's instructions to measure serum levels of adalimumab (Cat. No. 728552) and anti-adalimumab (Cat. No. 728533). All samples were measured in duplicate and repeated on 3 plates using a FLUOstar Omega Spectrophotometer (BMG Labtech) and MARS™ software. Pain related to the adalimumab injection was rated by the participant and the injection site assessed by visual inspection. Following surgery, the excised Dupuytren's tissue was transported to the lab, where the nodule was dissected to retain a central piece for histological examination whilst the remainder was frozen and pulverised. The resulting powder was split in two for protein or RNA extraction and stored at −80 °C. RNA was extracted and following the generation of cDNA, absolute levels of *ACTA2, COL1A1, COL3A1* and *CDH11* were determined using the standard curve method (appendix). Procollagen 1A1 protein levels were determined by DuoSet ELISA reagents (DY6220-05, R&D systems, Oxon, UK) in triplicate following the manufacturer's instructions. α-SMA protein levels were determined using a custom developed MSD^®^ plate (appendix 2). Tissue samples for histology were fixed in 4% paraformaldehyde, longitudinally bisected, embedded in paraffin wax and 7 μm sections obtained from the cut surface. Sequential sections were stained with hematoxylin-eosin, mouse anti–α-SMA antibody (Abcam 7817) or a mouse isotype control. Antibodies were detected using biotinylated anti-mouse antibody and avidin/biotin/HRP complex reagent (Vectastain ABC, Vector Lab, UK). Patients completed their standard care following surgery, returning at 2 weeks (±1 week) for assessment of the wound, change of dressing and commencement of hand therapy. Final clinical assessment was at 12 weeks (±4 weeks), when a photograph was obtained for final blinded assessment of wound healing and scarring.

### 2.3 Outcomes

The primary outcome was mRNA expression of *ACTA2* (α-SMA). Secondary outcomes comprised mRNA expression for *COL1A1, COL3A1* and *CDH11*, and protein levels of α-SMA. Protein levels of type I procollagen, type III procollagen, acid and pepsin soluble and insoluble collagen, alongside nodule hardness and size of the nodule on ultrasound scan were also assessed. Adverse events were assessed using visual inspection of the injection site and site of surgery, photographs and laboratory reports. Tertiary outcomes comprised circulating levels of adalimumab, antibodies to adalimumab and participant injection experience recorded as pain during injection using a 5-level Likert scale (1 = Not at all), and immediately after injection also using a Likert scale (1–10, 1 = no pain).

### 2.4 Randomisation and Masking

Participants were randomised for treatment with placebo or adalimumab using a 1:3 ratio with a block size of four. The dose cohort was determined by the progression of the trial, with initial patients allocated to the lowest dose cohort (15 mg adalimumab) and doses escalated (35 mg then 40 mg adalimumab) following a review of patient wound healing two weeks after surgery. The volume of saline solution for the placebo treatment matched the relevant volume associated with the dose cohort to ensure blinding. The allocation log, generated by the trial statistician, using the statistical software STATA version 13.0, was stored securely within the hospital pharmacy. Due to the distinctive packaging of the 40 mg in 0.4 ml preparation of adalimumab in pre-filled syringes, blinding of the person administering the injection was not feasible and therefore this healthcare professional was independent and not involved with routine patient care, recruitment, outcome assessment, surgery or follow-up. A shield prevented patients in this dose cohort from seeing the syringe. For all cohorts, participants, healthcare and laboratory professionals involved in recruitment, surgery, follow-up and outcome assessment were blinded to treatment allocation.

### 2.5 Statistical Analysis

This study was designed as a phase 2a clinical trial to enable estimation of potential dose and efficacy of adalimumab in inhibiting progression of DD. As no previous studies have explored the effect of adalimumab on *in vivo* expression of *ACTA2* mRNA, sample size was determined using *in vitro* findings and inflated to account for the non-clinical nature of the research upon which estimation was performed. Descriptive statistics, complemented using exploratory data analyses, were used to assess the demographics across intervention groups for each dose based on an intention-to-treat population. Additional analyses were performed using either linear mixed modelling or nested analysis of variance to account for trial structure, which included a random-effects component to allow for the values from three plates and duplicates per participant. Where appropriate, Tukey's HSD adjustment for multiplicity was applied when assessing contrasts. For the basis of most contrasts the patients allocated to placebo were pooled. The validity of samples for circulating levels of adalimumab could not be verified for three patients. To address this, a per-protocol approach was adopted and the corresponding patients were excluded from analysis.

### 2.6 Role of the Funding Source

The study was funded by the Health Innovation Challenge Fund (Wellcome Trust and Department of Health). Funding for purchase of the adalimumab was provided by 180 Therapeutics LLP. The funders had no involvement in study design or data analyses. The study was sponsored by the University of Oxford. Raw unblinded data were analysed by the trial statisticians (WS and SD) and subsequently made available to the remainder of the trial team. The corresponding author drafted the manuscript in conjunction with all the other authors and all share the responsibility for submission for publication.

## 3 Results

### 3.1 Patient Demographics

**Fig. 1:**
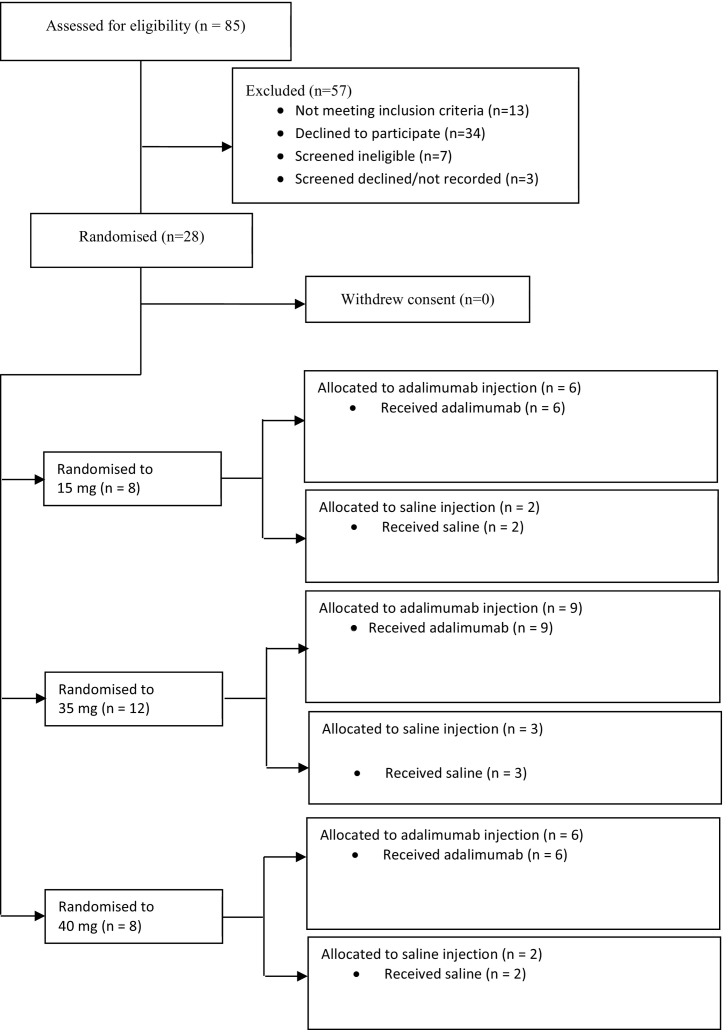
Trial profile.

Between November 2015 and November 2016, 85 participants were screened and 28 were randomised to successive dose escalation cohorts, 8 to the 15 mg adalimumab cohort, 12 to the 35 mg adalimumab cohort and 8 to the 40 mg adalimumab cohort. In each cohort, patients were randomised in a ratio of 3:1 to receive either adalimumab or an equivalent volume of placebo (saline) (Fig. 1). Baseline characteristics were similar between different cohorts (Table 1).

**Table 1:** Baseline characteristics for treatment groups. Age of onset of Dupuytren's disease and age on entry into trial shown by treatment group. Patients receiving placebo are pooled.

Treatment	Mean	SD	Min	Max	Median	n
Age upon onset (years)						
15 mg adalimumab	47.0	10.5	30	60	48.5	6
35 mg adalimumab	53.8	11.9	28	67	57.0	9
40 mg adalimumab	53.8	5.6	45	60	55.0	6
Sodium chloride 0.9%	56.1	8.5	42	69	57.0	7

Age upon trial entry						
15 mg adalimumab	57.2	9.1	48	71	53.0	6
35 mg adalimumab	63.7	9.9	48	78	64.0	9
40 mg adalimumab	63.3	5.7	56	69	65.0	6
Sodium chloride 0.9%	62.9	8.3	46	72	63.0	7

Total						
Age upon onset	52.9	9.8	28	69	56.0	28
Age upon trial entry	62.0	8.5	46	78	63.0	28

### 3.2 Outcomes

**Fig. 2:**
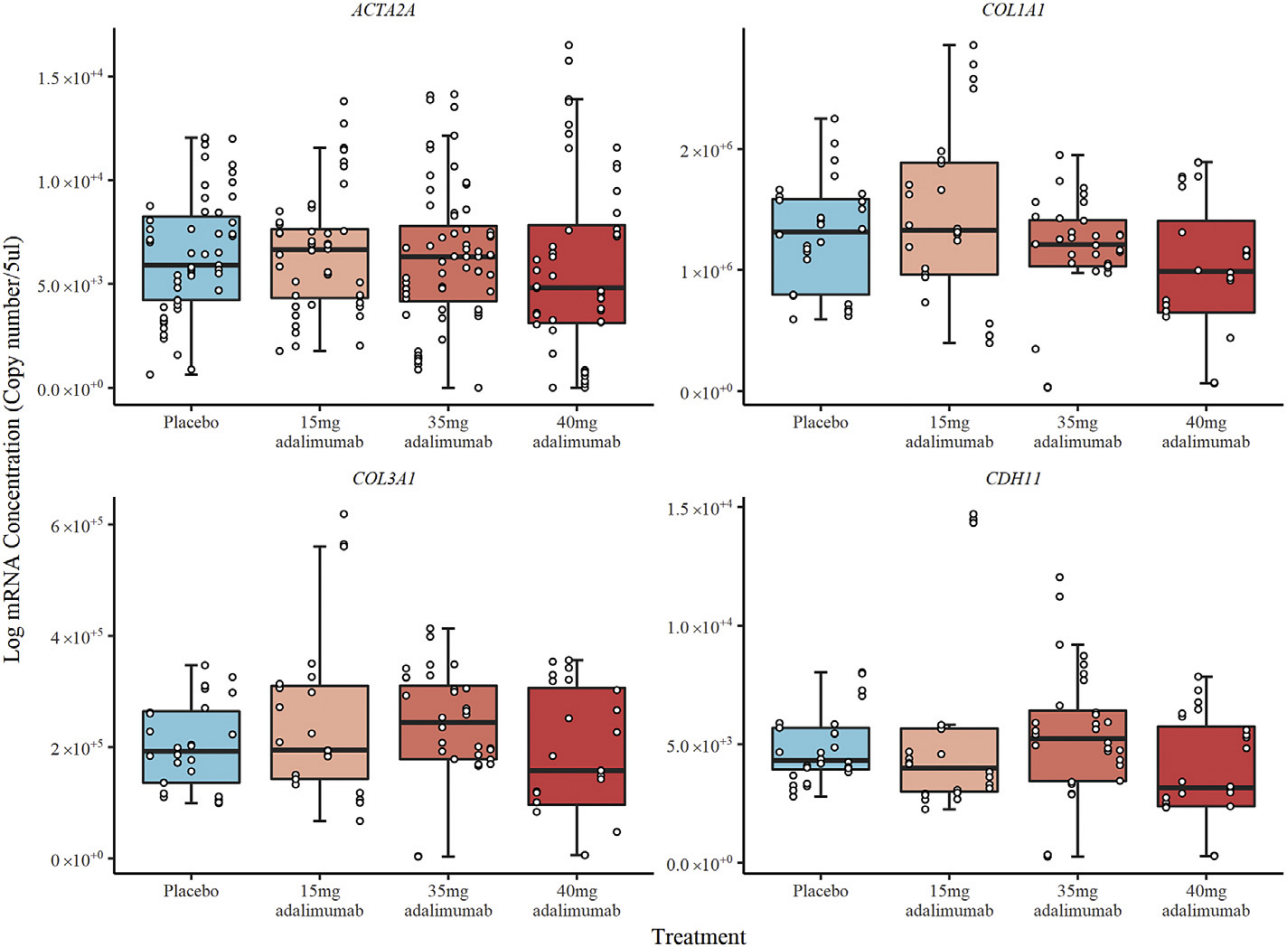
Box and whiskers plot of log RNA concentration by treatment received. The figure is divided into separate plots for each gene assessed. The box represents the inter-quartile range (IQR) and whiskers extend to 1.5 relevant IQR (Tukey boxplot). The x-axis shows individual patients, grouped by treatment received, with the three repeat measures performed for each patient represented by points stacked within the same column.

**Fig. 3:**
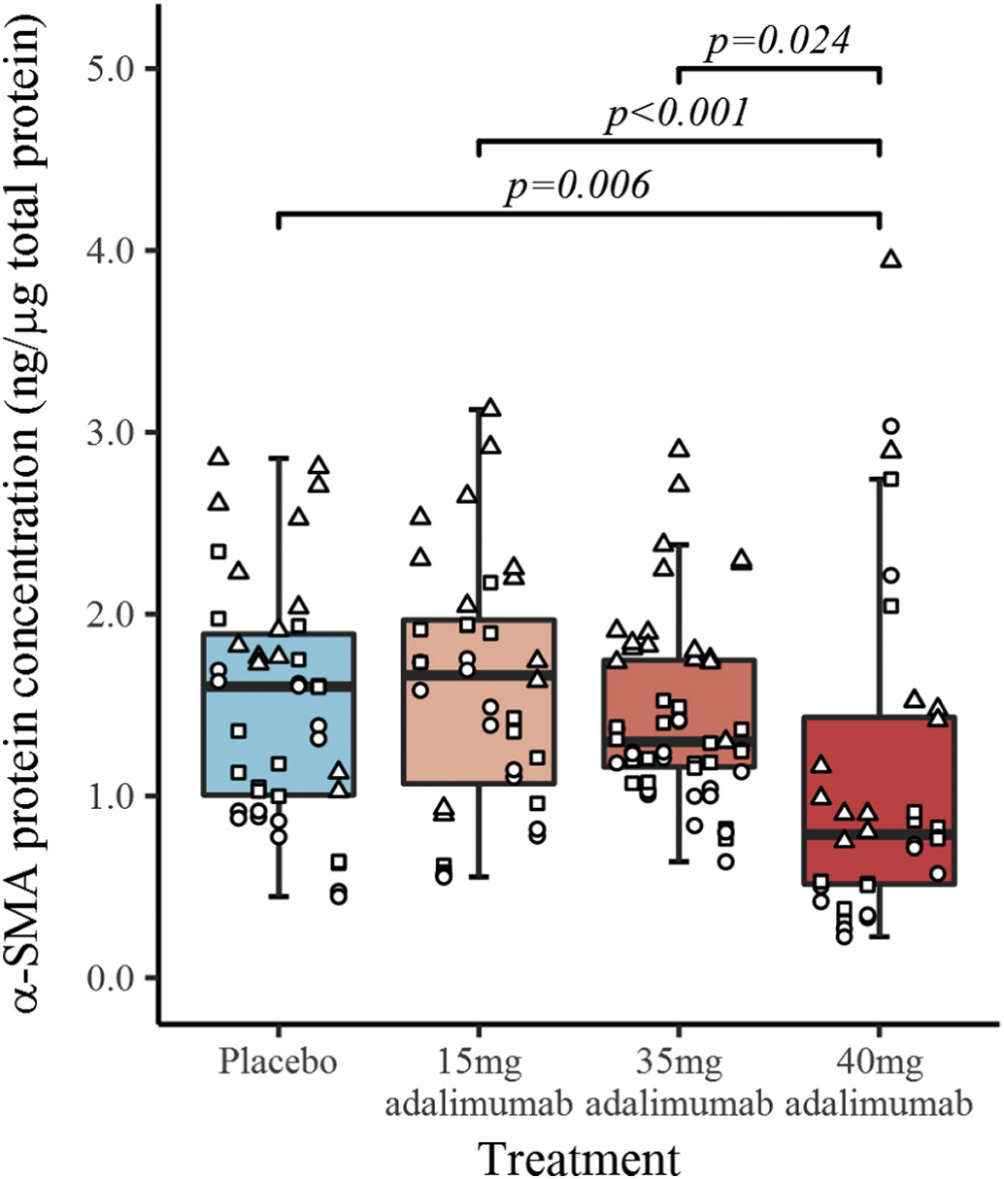
Box and whiskers plot of α-SMA protein concentration by treatment received. The box represents the inter-quartile range (IQR), the horizontal line represents the median and whiskers extend to 1.5 relevant IQR. The x-axis shows individual patients, grouped by treatment received. Samples from each patient were analysed in duplicate on three separate plates and the values for each plate are shown as circles, triangles or squares.

There was no difference between any of the groups, including placebo, in mRNA levels for genes that encode *ACTA2*, *COL1A1*, *COL3A1* or *CDH11* (Fig. 2). Levels of α-SMA protein expression in patients treated with 40 mg adalimumab (1.09 ± 0.09 ng per μg of total protein) were statistically significantly lower (*p* = 0.006) compared to placebo treated patients (1.51 ± 0.09 ng per μg of total protein) and compared to those treated with 15 mg (1.60 ± 0.09 ng per μg of total protein; *p* < 0.001) or 35 mg (1.44 ± 0.08 ng per μg of total protein; *p* = 0.024) adalimumab, whilst 35 mg and 15 mg adalimumab cohorts showed no difference compared to placebo (Fig. 3). Qualitative assessment of immunohistochemical staining for α-SMA showed reduced intensity for the responders in the 40 mg cohort compared to placebo (appendix, Supplementary Fig. 1).

**Fig. 4:**
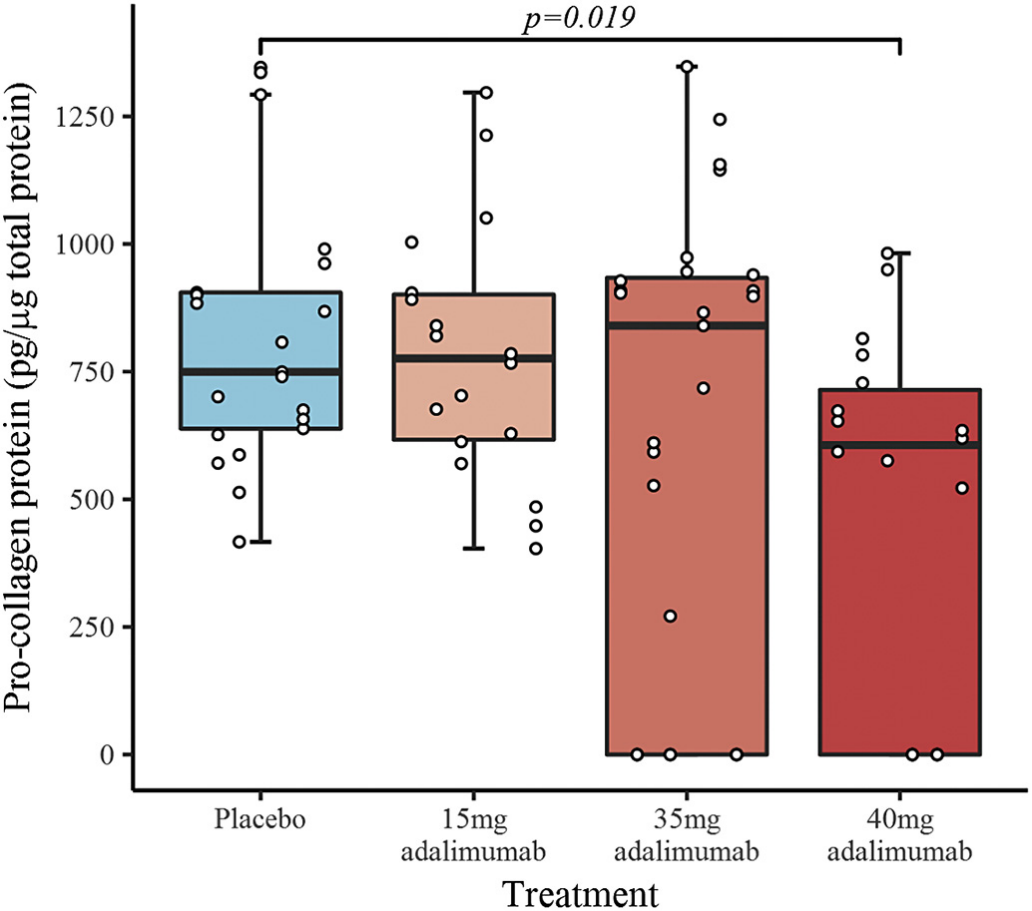
Box and whiskers plot of pro-collagen protein concentration by treatment received. The box represents the inter-quartile range (IQR), the horizontal line represents the median and whiskers extend to 1.5 relevant IQR (Tukey boxplot). The x-axis shows individual patients, grouped by treatment received, with the three repeat measures performed for each patient represented by points stacked within the same column. The plot includes pro-collagen concentrations reported below the minimum detectable range as zero, 3 of 9 in 35 mg and 2 of 6 in 40 mg adalimumab cohorts.

The levels of procollagen type I protein expression were significantly lower (*p* = 0.019) in the subgroup treated with 40 mg adalimumab (474 ± 84 pg/μg total protein) compared with placebo (817 ± 78 pg/μg total protein) (Fig. 4). Pro-collagen type I levels were below the minimum limit for quantification of 250 pg/μg in three patients injected 35 mg adalimumab and two injected with 40 mg adalimumab.

Tissue from nodules surgically excised two weeks after injection was also assessed for levels of procollagen type III and collagen using the Biocolor (UK) Sircol Soluble and Insoluble Collagen Assays. There were no differences between treatment groups and placebo for either assay.

Plasma levels of adalimumab and antibodies to adalimumab were assayed prior to intra-nodular injection of adalimumab and repeated 2 weeks (±3 days) after injection, prior to surgery. Prior to nodule injection, circulating blood adalimumab levels were below the minimum detectable limit for all patients. At two weeks after injection, patients within the placebo treatment group had undetectable levels of blood adalimumab whilst all patients within adalimumab treatment groups demonstrated detectable levels (appendix, Supplementary Table 1). Two weeks after treatment, no antibodies to adalimumab were detected amongst placebo treated patients, demonstrating assay specificity and the absence of cross reactivity. Antibodies were detected in one patient treated with 15 mg adalimumab, 5 patients treated with 35 mg adalimumab and two patients treated with 40 mg adalimumab. However, antibody levels only exceeded the minimum threshold for clinical significance [22] of 12 AU/ml in two of the three repeat measurements (mean±SD, 12.4±0.63 AU/ml) in one patient treated with 35 mg adalimumab.

Ultrasound scans performed prior to injection and two weeks later prior to surgery showed no significant changes in any of the dose cohorts in nodule size based on the area of the nodule, the length of the perimeter and the feret (maximum length across the nodule) (appendix, Supplementary Fig. 2). Similarly, there was no change in nodule hardness measured using a durometer in any of the dose cohorts when comparing before and two weeks after injection (appendix, Supplementary Fig. 3).

All but one patient reported pain during the injection, with higher levels of pain experienced with larger volumes and with the more dilute formulation of adalimumab. The Likert scores immediately after injection showed that high volume of injection (0.7 ml) was associated with more pain than lower volumes (0.3–0.4 ml) and that the 40 mg in 0.4 ml adalimumab formulation was associated with lower pain scores (appendix, Supplementary Fig. 4).

### 3.3 Adverse Events

Two patients suffered serious adverse events (SAE) after injection. One patient fell two months before injection of placebo whilst running and presented 5 days after surgery for DD with impaired motor function due to bilateral subdural haematomas that were treated non-operatively. He made a full recovery with spontaneous resolution of the haematomas. A patient with type 2 diabetes who received an injection of 35 mg of adalimumab into a nodule situated over the proximal phalanx of the little finger presented four days after surgical fasciectomy with a suture abscess at the distal palmar crease in the axis of the ring finger. He was initially treated with oral antibiotics by his general practitioner but due to lack of improvement after 5 days he was admitted to hospital for i.v. antibiotics. Inspection of the wounds revealed erythema around the sutures and a small amount of pus was expressed following suture removal. He was discharged home the following day on oral antibiotics and the wound healed uneventfully. Blinded assessment of photographs of scars obtained 12 weeks (±4 weeks) after surgery showed well-healed mature scars in all patients except the patient who developed the post-operative wound infection, where the scars were more prominent. Both events were considered by the local investigator and an independent reviewer not to be related to the investigational medicinal product and the second SAE was considered to be related to the surgical procedure.

Swelling at the injection site was only apparent in 3 patients receiving 35 mg adalimumab and two receiving 0.7 ml saline. Transient redness was noted at the injection site in 7 patients distributed across the different treatment groups. No patient experienced blistering, haematoma, bruising, local itching or signs of nerve injury. No patient reported any adverse events one week after injection.

## 4 Discussion

Our results show that two weeks following administration of 40 mg of adalimumab in 0.4 ml into Dupuytren's nodules there was down regulation of the myofibroblast phenotype as evidenced by lower expression of α-SMA and pro-collagen type I proteins. These findings represent the clinical translation of our *in vitro* data based on human tissue where we showed that tumour necrosis factor (TNF) selectively converts precursor palmar fibroblasts from Dupuytren's patients to myofibroblasts *via* the Wnt signalling pathway, and anti-TNF is inhibitory [18]. Like all fibrotic conditions, Dupuytren's disease is characterised by the deposition of excessive collagenous extracellular matrix which is remodelled and contracted by α-SMA-expressing myofibroblasts [15], that aggregate in nodules [17]. Collagen is synthesised and secreted in the precursor state as procollagen and the N- and C-terminal globular domains are cleaved at the plasma membrane to produce tropocollagen that self-aggregates to form fibrils, which grow and are eventually cross-linked by lysyl oxidase to form insoluble collagen [23]. The soluble Sircol assay detects all the uncross-linked forms and the insoluble assay only detects the cross-linked collagens. The short half-life of procollagen due to its rapid conversion to fibrillar collagen may explain the reduction in type I procollagen we observed at the two-week primary endpoint and it is possible that soluble and insoluble collagens may also change but at later time points. Although type III collagen comprises 35–49% of the total [24] in Dupuytren's nodules, which represent the earlier stages of disease, we were unable to detect any changes in type III procollagen due to the poor sensitivity of the only commercially available assay, with levels falling below the threshold of detection in half of samples in all treatment groups. The downregulation of both procollagen and the contractile protein α-SMA by local inhibition of tumour necrosis factor (TNF) provides the first successful proof of concept for targeted treatment of the pathological mechanisms underlying this common localised fibrotic disease of the hand that affects 4% of the general UK and US populations [1].

There was no change in the levels mRNA levels for genes that encode for *ACTA2*, *COL1A1*, *COL3A1* and *CDH11*. The most likely explanation for the down regulation in protein expression for α-SMA and type I procollagen without a concomitant change in the respective mRNA is that expression of these proteins is predominantly regulated at the post-transcriptional level. We have previously shown to be the case for α-SMA in Dupuytren's myofibroblasts [25], but did not select α-SMA protein levels as the primary outcome measure as the assay for the protein was optimised over the course of the trial. Collagen 1 expression is also mainly regulated post translationally in fibrotic conditions [26]. Another potential but less likely explanation would be different half-lives of mRNA and collagen proteins of hours and days respectively [26], which would potentially allow mRNA levels to recover at 2 weeks whilst corresponding protein levels remained suppressed.

The downregulation of α-SMA and procollagen type I only occurred in patients where 40 mg of adalimumab in 0.4 ml was injected directly into the nodule, but not following administration of 35 mg in 0.7 ml. This could be explained by some of the adalimumab in the more dilute preparation extravasating out of the nodule and hence not available to act at high concentrations locally on the aggregates of myofibroblasts and immune cells. An alternative but less likely explanation is that the excipients in the more dilute preparation of adalimumab rendered it less efficacious for this application. Our data would suggest that only high local concentrations of adalimumab are effective in down regulating the myofibroblast phenotype in DD and it is unlikely adalimumab or other anti-TNF drugs would be efficacious if administered systemically. This would be consistent with the low-grade inflammation that is confined to the nodules of DD [18]. The half-life of adalimumab is 2 weeks [27], and trough levels at this time for patients on regular systemic therapy of 40 mg every 2 weeks averaged ~5μg/ml in patients with rheumatoid arthritis and 6–10μg/ml in patients with psoriatic arthropathy not treated with concomitant methotrexate [27]. This would suggest that the kinetics of absorption of adalimumab when injected into the nodules of DD may be similar to when the drug is administered subcutaneously. We would postulate that formulations that retard dissipation of adalimumab from the site of injection are likely to be more effective and may need to be administered less frequently for the treatment of localised fibrotic conditions characterised by low-grade inflammation such as DD. Neutralising antibodies to adalimumab are likely to result in reduced efficacy [28]. Only one patient, who received 35 mg in 0.7 ml, had antibody levels (12.4±0.63 AU/ml) just above the threshold of significance of 12Au/ml [22], which are unlikely to be of clinical significance. Neutralising antibodies may occur more commonly after repeated administration of adalimumab, especially in patients who are not on concomitant methotrexate.

Whilst almost all patients reported pain during injection, pain scores immediately after injection were higher in participants receiving volumes of either placebo or adalimumab >0.3–0.4 ml and in those injected with the formulation of adalimumab comprising 40 mg in 0.8 ml of carrier that was used for the 15 mg and 35 mg cohorts. This formulation differs significantly from the more concentrated form of 40 mg in 0.4 ml in that it contains a variety of excipients [27], including citrate, which is associated with higher pain scores when injected subcutaneously [29]. The 40 mg in 0.4 ml formulation was not available commercially when the trial opened to recruitment and therefore the study was initially based on dose cohorts using the 40 mg in 0.8 ml formulation. Transient swelling at the injection site was apparent in some patients receiving 0.7 ml of either placebo or adalimumab, most likely due to extravasation of the injected material outside the confines of the nodule. The safety profile of adalimumab is well-known and the commonest adverse events in patients with autoimmune disorders on long-term therapy are related to infection [30]. One patient in our study, who suffered from type 2 diabetes mellitus received 35 mg of adalimumab developed a wound infection 4 days following surgery that resolved following removal of sutures and antibiotics. The infection was considered to be unrelated to the adalimumab injection as it was a localised suture abscess away from the site of the injected nodule. Wound infection has been reported to occur in approximately 3.6% of patients undergoing Dupuytren's fasciectomy [8].

Unsurprisingly, there was no difference in nodule hardness or size as assessed by tonometry and ultrasound scan respectively at 2 weeks post injection. This trial enabled us to optimise the methods for obtaining the data and analysing them in preparation for follow up studies.

## 5 Conclusions

This phase 2a randomised trial shows that a single intranodular injection of 40 mg adalimumab in 0.4 ml in patients with Dupuytren's disease is safe and leads to down regulation of the myofibroblast phenotype as evidenced by reduced expression of α-SMA and type I procollagen proteins. Having defined the most efficacious dose and preparation and based on these positive proof of concept data we are now proceeding with a phase 2b trial in 138 patients with early stage Dupuytren's disease randomised 1:1 to receive 4 injections of adalimumab or placebo at 3 month intervals and followed for a total of 18 months from baseline [21]. Our study also illustrates the utility of using early stage fibrotic human tissue to elucidate novel therapeutic targets [18] that can be translated to the clinic.

## Conflicts of Interest

Mrs. Ball, Dr. Davidson, Dr. Williams, Dr. Sones, Dr. McCann, Ms. Cabrita, Dr. Swettenham, Dr. Cahoon, Dr. Copsey, Dr. Francis, Dr. Black, Dr. Barber, Mrs. Dutton report grants from Wellcome Trust, grants from Department of Health, other from 180 Therapeutics LP, during the conduct of the study.

Professor Taylor reports grants from Wellcome Trust, grants from Department of Health, other from 180 Therapeutics LP, during the conduct of the study; grants from UCB, personal fees from UCB, AbbVie, Pfizer, outside the submitted work.

Professor Lamb reports grants from Wellcome Trust, grants from Department of Health, during the conduct of the study; Professor Sarah Lamb reports grants from NIHR Health Technology Assessment Programme during the conduct of this study.

Professor Nanchahal and Professor Feldmann report grants from Wellcome Trust, grants from Department of Health, other from 180 Therapeutics LP, during the conduct of the study; In addition, Professor Nanchahal has a patent PCT/EP2011/069147 issued to 180 Therapeutics, and with Professor Feldmann a patent PCT/US2017/049691 pending, a patent PCT/US2017/026382 pending, a patent 16759325.0 EP2017 pending, and a patent 16759326.8 EP2017 pending.

## Funding Sources

We would like to acknowledge funding from the Health Innovation Challenge Fund (HICF) (HICF-R8-433), a parallel funding partnership between the Wellcome Trust and the Department of Health, 180 Therapeutics, and the National Institute for Health Research (NIHR) Oxford Biomedical Research Centre (BRC). The views expressed are those of the authors and not necessarily those of the NHS, the NIHR or the Department of Health.

## Author Contributions

JN, SL, MF, CB, SD and PT conceived and designed the study. DD and NC recruited the patients. CB, DD and NC collected the clinical data. LW, FM and MC performed the laboratory analyses. WS, SD and BC performed the statistical analyses. JS, VB, JB, EAF and SL managed the trial.

## Appendix A Supplementary Data

Supplementary material Image 1
