# Supplementary material for: Anti-Tumour Necrosis Factor Therapy for Dupuytren's Disease: A Randomised Dose Response Proof of Concept Phase 2a Clinical Trial
Source: eBioMedicine. 2018 Jul 6;33:282–8. doi: 10.1016/j.ebiom.2018.06.022 (PMC6085556; doi:10.1016/j.ebiom.2018.06.022)
Supplement: Supplementary file 1 — Supplementary material [file mmc1.docx]

**Supplementary Appendix**

Content

1. Supplementary methods
2. Supplementary results
3. References

**Supplementary Methods**

***S1 Pro-COL1A1 and α-SMA quantitation***

Tissue samples were initially cut into small pieces (~3-5mm) and immediately snap frozen in liquid nitrogen. Whilst maintaining all samples and equipment on dry ice, samples were pulverized using BioSpec Bio-pulveriser, (Bartlesville, OK, USA).  The Bio-pulverizer quickly fragments hard frozen tissue samples into tiny pieces. Protein was extracted using RIPA buffer containing protease inhibitor cocktail (Sigma, UK) from 10mg of pulverised tissue powder using Homogeniser (Ultra-Turrax T8, IKA-Werke) at setting 5, to pulse for 2 seconds, 6 times. Samples were lysed for 20 min, followed by centrifugation (10mins, 13,000 rpm, 4^o^C) and supernatants were collected and stored at -80^o^C prior to analysis. Protein levels were determined using BCA Protein assay (Thermo Scientific,UK) and quantified using FLUOstar Omega Spectrophotometer (BMG Labtech) and MARS™ software. Using Abnova (Taipei City, Taiwan) matched anti-α-SMA antibody pairs (H00000059-AP41), a standard bind MSD plate (Rockville, MD) was coated with capture antibody (1/180) 25μl/well diluted in PBS and left at 4^o^C overnight on a shaker. Plates were washed 3 times with PBST and blocked with 5% milk powder (Marvel™) in PBS (PBST) for 2 hr, then washed 3 times in PBST. Standard curve; human recombinant α−SMA (ACTA2) (LSBio, Seattle, WA) was applied to the plate in duplicate; standard curve 500 ng/ml – 8ng/ml in, 3 fold serial dilutions (in diluent of 2% Milk PBST). Trial samples (0.5µg) were added in duplicate, repeated in 3 plates and left overnight at 4^o^C shaking. Plates were washed 3 times in PBST and detection antibody was added (1/450) in diluent and left for 2 hr at room temp, shaking, then washed 3 times in PBST. MSD^®^ sulfo-tag (Rockville, MD) was added (1/1000), and left for a further hour, shaking at room temperature, then washed 3 times in PBST prior to the addition of 2X MSD^®^ read buffer (diluted in dH_2_0). Plates were read immediately on MSD® SECTOR Imager 6000 using MSD^®^ software to determine α-SMA concentration (ng/ml) from standard curve. A standard curve was generated by the software. If the standard curve exceeded R^2^≥0.98 the data was deemed to have passed QC.

***S2 Quantitative PCR***

Standards were sourced as purified plasmids from *PGK1* NM_004048 clone ID SC117632 and *B2M* NM_000291 clone ID SC119988 from Origen (Rockville, MD, USA), *ACTA2* NM_001141945.2 Clone ID 108H05, *Cadherin-11* NM_001797.3 Clone ID D017D, *COL1A1* NM_ 000088 Clone ID 31G04, *COL3A1*, NM_000090 Clone ID 50119, *GAPDH* NM_00204 Clone ID G036D from Source BioScience (Cambridge UK). 5ng of purified plasmids were linearised with; *ACTA2* SFiI, (Fermentas, Thermo Fisher, UK), *B2M HindIII* (Fermentas), *CDH11, COL1A1 and COL3A1,* *Not1* (Promega, UK) and *PGK1 EcoRI* (Fermentas). DNA was quantified using Nanodrop™ Spectrometer and diluted such that 5µl contained 10,000,000 copies of each gene and a 6 point (1:10 dilution) standard curve was generated. 10µl PCR reactions were performed using 5µl of cDNA, 0.05µl of Qiagen Quantitect primers (Hs_ ACTA2_SG Cat No. QT00088102), (Hs_COL1A1_SG Cat No. QT 00037793), (Hs_COL3A1_SG Cat No. QT000058233), (Hs_B2M_1_SG QT00088935), (Hs_PGK1_1_SG Cat No. QT00013776) and GAPDH Invitrogen–Life Technologies (For) GAAGGTTGAAGGTCGGAGTC, (Rev) GAAGATGGTGATGGGATTTC and PCR 2X Syber Green Master Mix (Life Technologies, UK). All standards and trial sample cDNA were set up in quadruplicate on the *ViiA7*™ Real- Time PCR System, Applied Biosystems.

***Interpolation of Results***

Number of copies of each gene of interest were calculated from each corresponding standard curve using *Vii*A7 software. The value corresponding to the number (No.) of copies of each gene of interest; *ACTA2/ COL1A1, COL3A1 or CHD11* was normalised using the geometric mean of 3 different housekeeping genes (*GAPDH, PGK1,B2M*) as described (Vandesompele et al., 2002). If the standard curve exceeded R^2^≥0.98 the data was deemed to have passed QC.

= No. of Copies of *ACTA2/COL1A1/COl3A1/CDH11*

(No. of Copies of *GAPDH* x No. of Copies of *PGK1* x No. of Copies of *B2M*)^1/3^.

**Supplementary Results**


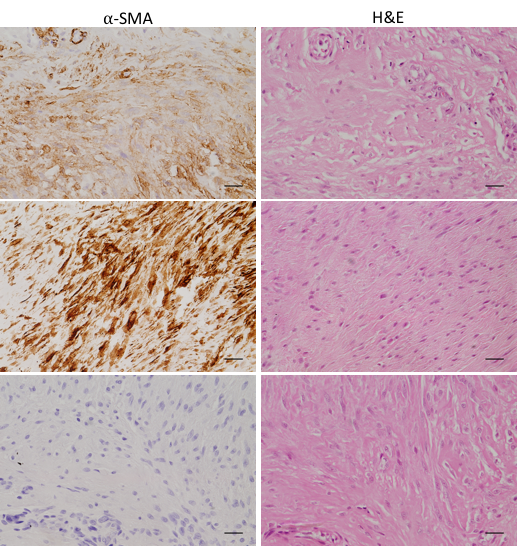


A

C

B

***Supplementary figure 1.* Representative images of immunohistochemical staining for α-SMA and corresponding serial sections stained with H&E. (A) 40mg adalimumab in 0.4ml carrier (B) 0.4ml placebo and (C) nodule stained with control murine isotype antibody. The reduced intracellular staining intensity in the myofibroblasts in (A) compared to placebo (B) is consistent with the decrease in α-SMA protein observed in the 40mg adalimumab dose group (Fig 2). Scale bar 25μm.**

| ***Supplementary table 2.* Circulating blood adalimumab at two weeks post-treatment** | | | | |
| --- | --- | --- | --- | --- |
| Treatment | Samples | Patients | Mean (μg/ml) | SD (μg/ml) |
| Placebo | 24 | 6 | 0* | 0* |
| 15mg adalimumab | 16 | 4 | 2.11 | 0.36 |
| 35mg adalimumab | 36 | 9 | 3.13 | 0.86 |
| 40mg adalimumab | 24 | 6 | 2.91 | 0.55 |
| *Below minimum detectable limit | | | | |

**
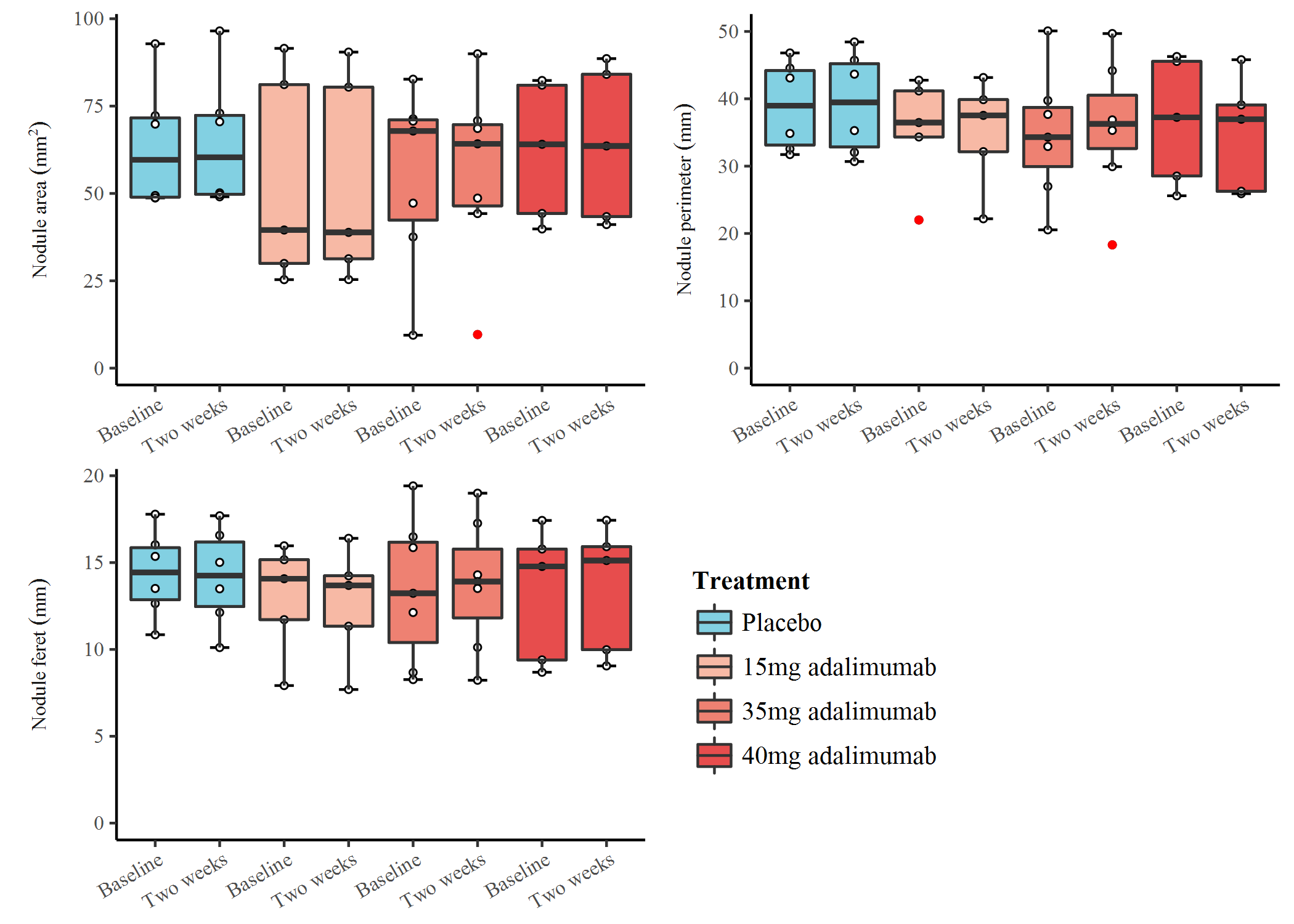
**

***Supplementary figure 2:* Box and whiskers plot of nodule area, perimeter and maximal feret on ultrasound scan by treatment received. The box represents the inter-quartile range (IQR), the horizontal line represents the median and whiskers extend to 1.5 relevant IQR (Tukey boxplot). Values determined for each patient are represented using points. Scans from 1 patient injected with placebo, 1 patient in the 15mg cohort, 2 in the 35mg cohort and 1 in the 40mg cohort could not be analysed due to a faulty ultrasound probe.**

**
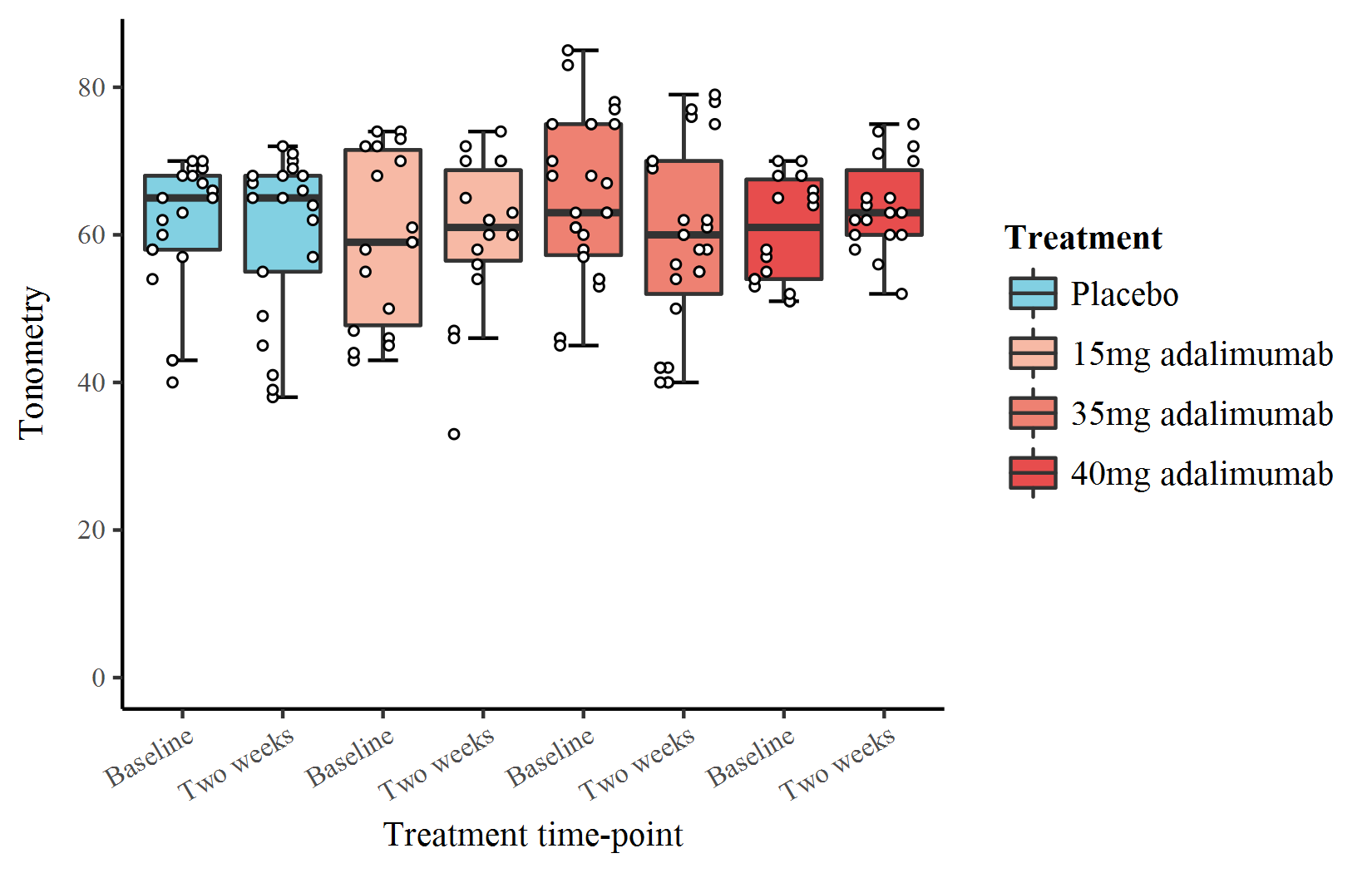
**

***Supplementary figure 3:* Box and whiskers plot of nodule hardness assessed by tonometry by treatment received. The box represents the inter-quartile range (IQR), the horizontal line represents the median and whiskers extend to 1.5 relevant IQR (Tukey boxplot). The x-axis shows individual patients, grouped by treatment received, with the three repeat measures performed for each patient represented by points stacked within the same column.**


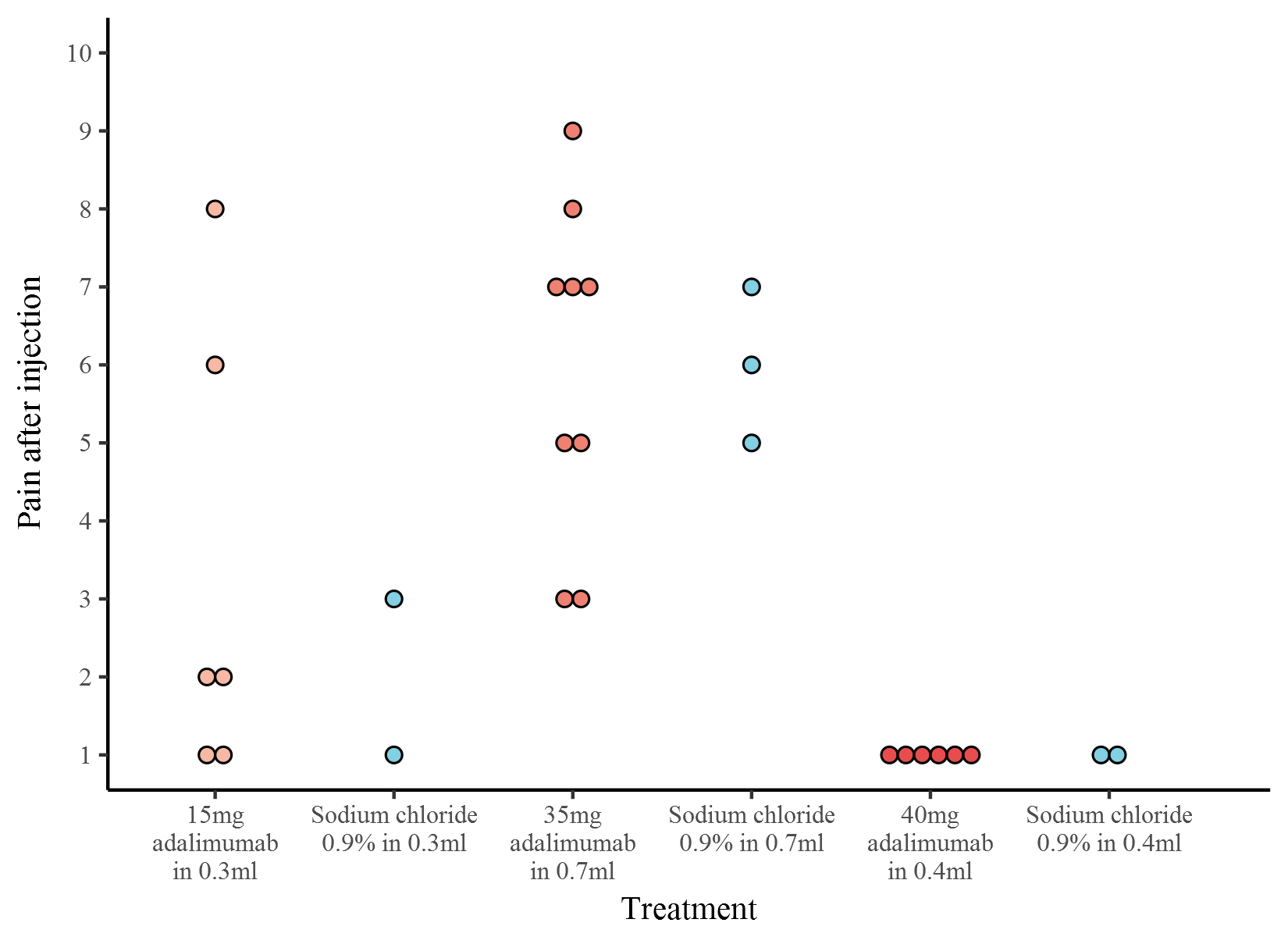


***Supplementary figure 4.* Scatterplot of pain experienced by each patient immediately following injection. Adalimumab shown in red, placebo in blue.**

**Supplementary Reference**

Vandesompele, J., De Preter, K., Pattyn, F., Poppe, B., Van Roy, N., De Paepe, A., and Speleman, F. (2002). Accurate normalization of real-time quantitative RT-PCR data by geometric averaging of multiple internal control genes. Genome Biol *3*, RESEARCH0034.
